# P-1452. Exploration of Associations Between Social Determinants of Health and Treatment Failure in People with HIV

**DOI:** 10.1093/ofid/ofae631.1624

**Published:** 2025-01-29

**Authors:** Smitha Gudipati, Victoria Warzocha, Indira Brar, Dwayne Baxa, Ramin Homayouni, Norman Markowitz

**Affiliations:** Henry Ford Health System, Detroit, Michigan; Henry Ford Health, Detroi, Michigan; Henry Ford Hospital, Detroit, Michigan; Oakland University, Detroit, Michigan; Oakland University, Detroit, Michigan; Henry Ford Health System, Detroit, Michigan

## Abstract

**Background:**

Addressing Social Determinants of Health (SDOH) and health disparities is at the core of “Ending the HIV Epidemic.” To address SDOH, there is a funded HIV Care Coordination Program (CCP) at Henry Ford Health (HFH), with support to surmount SDOH in people with HIV (PWH). In this pilot study, we explore associations between SDOH and treatment failure (TF) in this program.

**Figure:**
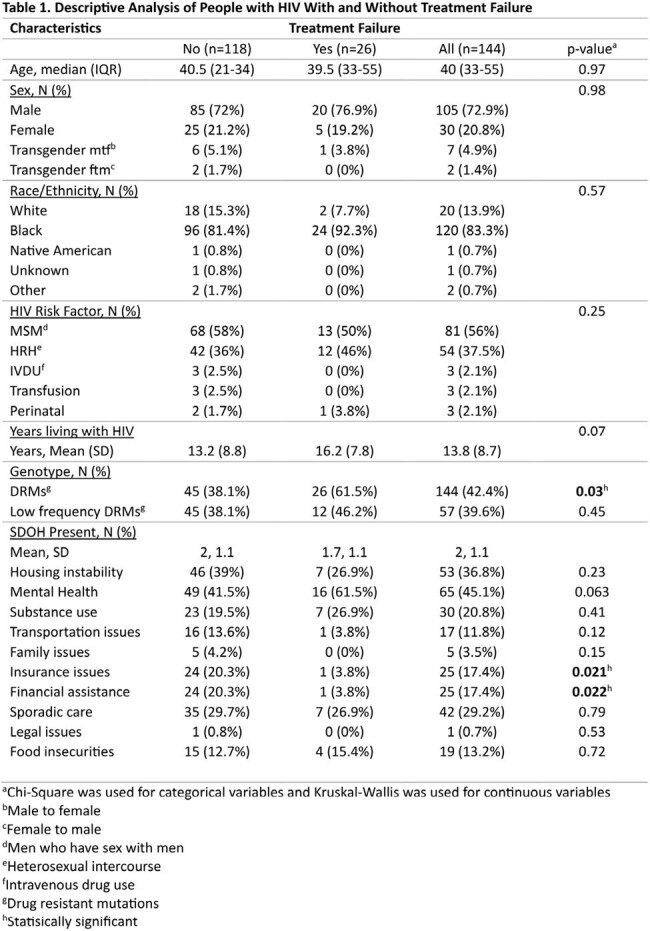

**Methods:**

A retrospective study was conducted on PWH in our CCP at HFH from 2022 to 2023. Variables were extracted from data monitoring. CCP enrollment required referral by HIV providers. PWH were surveyed on SDOH related to ART challenges, and these SDOH were assessed throughout the course of one year. HIV genotypes were obtained upon enrollment, and HIV-1 viral load (VL) was collected every 3 months for one year. Significant differences between PWH with or without TF (defined as VL > 200 copies/mL at 1 year) were determined using Chi-Square test for categorical variables and Kruskal-Wallis for continuous variables. Multivariable logistic regression was used to find associations between independent variables and TF.

**Results:**

A total of 144 PWH were included in this study and divided into with (n=26) and without (n=118) TF (Table 1). There were no statistically significant differences in age, gender, risk factors and mean years living with HIV between the two groups. TF PWH had significantly lower incidence of insurance issues and need for financial assistance. Using linear regression analysis while adjusting for age, race, gender, genotype and other SDOH factors, there was no significant association between insurance issues or financial assistance. Although drug resistant mutations (DRMs) were not linked to specific demographics or SDOH, TF PWH had a significantly higher number of DRMs at baseline (p< 0.0176, OR 3.45, 95% CI 1.24-9.60) and a trend towards increased low frequency (< 10%) DRMs.

**Conclusion:**

TF occurred in 18% of PWH with adverse SDOH at enrollment into CCP. While we were unable to identify an association of specific SDOH with TF, the TF rate was elevated in these PWH. Overcoming SDOH is essential to achieve viral suppression. DRMs at CCP entry were significantly associated with TF, and were present in all those with TF. Our study is ongoing, and with larger numbers, we aim to assess the impact of SDOH in TF PWH in future studies.

**Disclosures:**

**Indira Brar, MD**, Gilead: Advisor/Consultant|Gilead: Grant/Research Support|Gilead: Honoraria|Merck: Grant/Research Support|ViiV Healthcare: Grant/Research Support|ViiV Healthcare: Honoraria

